# MicroRNA-30 inhibits neointimal hyperplasia by targeting Ca^2+^/calmodulin-dependent protein kinase IIδ (CaMKIIδ)

**DOI:** 10.1038/srep26166

**Published:** 2016-05-20

**Authors:** Yong Feng Liu, Amy Spinelli, Li-Yan Sun, Miao Jiang, Diane V. Singer, Roman Ginnan, Fatima Z. Saddouk, Dee Van Riper, Harold A. Singer

**Affiliations:** 1 From the Center for Cardiovascular Sciences, Albany Medical College, Albany, NY (USA)

## Abstract

The multifunctional Ca^2+^/calmodulin-dependent protein kinase II δ-isoform (CaMKIIδ) promotes vascular smooth muscle (VSM) proliferation, migration, and injury-induced vascular wall neointima formation. The objective of this study was to test if microRNA-30 (miR-30) family members are endogenous regulators of CaMKIIδ expression following vascular injury and whether ectopic expression of miR-30 can inhibit CaMKIIδ-dependent VSM cell function and neointimal VSM hyperplasia induced by vascular injury. The CaMKIIδ 3′UTR contains a consensus miR-30 binding sequence that is highly conserved across species. A significant decrease in miR-30 family members and increase in CaMKIIδ_2_ protein expression, with no change in CaMKIIδ mRNA expression, was observed in medial layers of VSM 7 days post-injury. *In vitro*, overexpression of miR-30c or miR-30e inhibited CaMKIIδ_2_ protein expression by ~50% in cultured rat aortic VSM cells, and inhibited VSM cell proliferation and migration. *In vivo*, lenti-viral delivery of miR-30c into injured rat carotid arteries prevented the injury-induced increase in CaMKIIδ_2_. Furthermore, neointima formation was dramatically inhibited by lenti-viral delivery of miR-30c in the injured medial smooth muscle. These studies define a novel mechanism for regulating CaMKIIδ expression in VSM and provide a new potential therapeutic strategy to reduce progression of vascular proliferative diseases, including atherosclerosis and restenosis.

Vascular smooth muscle (VSM) cells are not terminally differentiated^1^,^2^ and undergo phenotype dynamics during development, wound healing and some occlusive vascular diseases including atherosclerosis, post-angioplasty restenosis, vein graft failure and arteriovenous fistula failure^3^,^4^. De-differentiated or “synthetic phenotype” VSM cells gain the features of enhanced proliferation and migration, and loss of contractile smooth muscle maker genes. Better understanding of signals and mechanisms involved in VSM cell phenotype dynamics will further advance the knowledge of initiation and progression of these vascular diseases and provide potential targets for disease treatment and prevention.

Multifunctional Ca^2+^/calmodulin-dependent protein kinase II (CaMKII) is an important mediator of Ca^2+^ and reactive oxygen species signals that regulate VSM function including cell proliferation and migration^5^. Mammalian CaMKII is encoded by four homologous and highly conserved genes (α, β, γ and δ) which organize into large hetero-multimeric holoenzymes^5^,^6^. While CaMKIIα and −β isoforms are most highly expressed in neural tissues^7^, CaMKIIδ and –γ are relatively ubiquitous and are the predominant isoforms expressed in VSM^8^,^9^. VSM phenotype switching is associated with reciprocal changes in CaMKIIδ and −γ isoforms^10^,^11^ with important functional consequences. CaMKIIδ expression is significantly up-regulated during VSM phenotype switching^10^,^12^, and silencing CaMKIIδ expression *in vitro* using siRNA inhibits VSM cell proliferation and migration^10^. Molecular and genetic approaches in rodent models indicate that CaMKIIδ is required for injury-induced vascular remodeling and neointimal hyperplasia^5^. Conversely, CaMKIIγ expression is down-regulated with VSM cell culture or arterial injury and appears to function in opposition to CaMKIIδ^11^. Mechanisms regulating CaMKIIδ or −γ expression dynamics in VSM are unknown.

MicroRNAs are small non-coding RNAs approximately 22 nucleotides in length. Mammalian miRNA genes are transcribed by RNA polymerase II in the nucleus, and undergo nuclear cleavage by Drosha, liberating a 60–70 nt miRNA precursor^13^,^14^. Pre-miRNA is actively transported from the nucleus to the cytoplasm by Ran-GTP^15^,^16^ and it is further processed by Dicer in the cytoplasm, forming mature miRNA (~22 nt)^17^. Mature miRNA functions via base-pairing with complementary sequences within target mRNA, which recruits AgoII to mRNA forming RISC (RNA induced silencing complex) and resulting in either mRNA degradation or translation suppression^18^. So far, miRNAs have been reported to regulate diverse cellular functions, including differentiation, proliferation, migration, apoptosis, cell cycle, *etc*. in various systems, such as tumor cells and cardiomyocytes^18^,^19^. Several studies have implicated regulation of CaMKIIγ expression by miR-219 in brain^20^,^21^. In VSM, Dicer knockout results in increased CaMKIIδ expression^22^ and a function for miR143/145 has been suggested, either directly by regulating CaMKIIδ mRNA translation, or indirectly by regulating VSM phenotypic modulation by targeting a network of transcription factors, including Klf4, myocardin and Elk-1^23^. Both miR-145^24^ and miR-30b^25^ have been shown to regulate CaMKIIδ expression in cardiomyocytes. Thus, microRNA-dependent mechanisms may be key for regulating CaMKIIδ or −γ expression dynamics physiologically and pathophysiologically.

In this study, we evaluated miR-30 family members as potential regulators of CaMKIIδ expression and CaMKIIδ-dependent function associated with phenotype switching in VSM. Expression of five miR-30 family members were strongly down-regulated in de-differentiated cultured VSM cells compared to differentiated medial wall VSM. Overexpression of miR-30 in cultured VSM significantly inhibited CaMKIIδ protein expression by targeting CaMKIIδ mRNA 3′UTR. Overexpression of miR-30 impaired VSMC proliferation which could be partially rescued by ectopic CaMKIIδ re-expression. Moreover, miR-30 family members were significantly downregulated in the vascular wall in response to vascular injury with coincident up-regulation of CaMKIIδ protein expression. Lenti-viral rescue of miR-30 in the injured arteries significantly prevented the increase in CaMKIIδ expression and reduced VSM cell proliferation and neointimal formation. Taken together, these studies establish negative regulation of CaMKIIδ by miR-30 in VSM, and indicate that decreased miR-30 expression associated with VSM phenotype switching is a potential mechanism for regulating vascular remodeling in response to injury.

## Results

### CaMKIIδ_2_ protein expression, but not mRNA, is rapidly upregulated in injured carotid arteries

**Figure 1:**
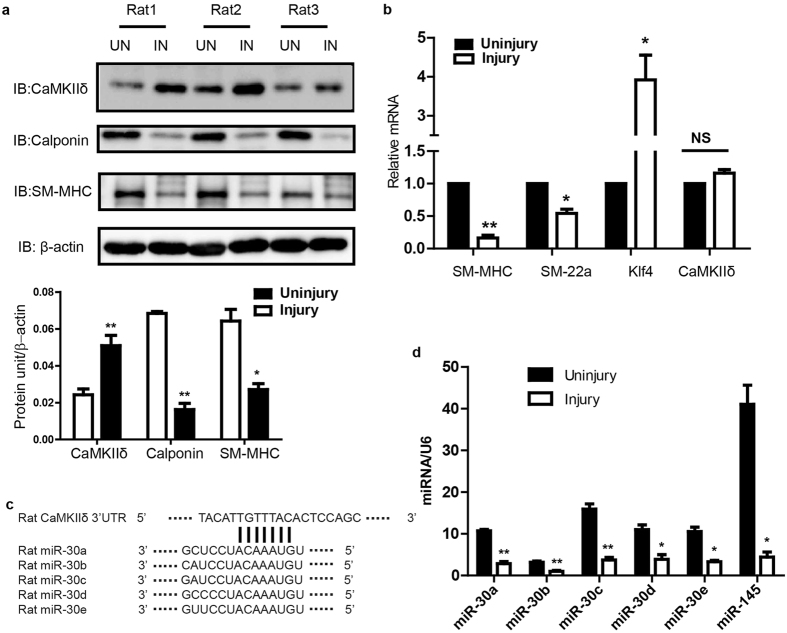
Vascular injury induces reciprocal regulation of miR-30 family members and CaMKIIδ protein in rat carotid artery. (**a**) Rat carotid artery was intraluminally injured and the protein levels of CaMKIIδ, Calponin, SM-MHC and β-actin (as loading reference) were measured by SDS-PAGE and immnuno bloting using specific antibodies. Histogram shows the quantification of protein levels normalized over β-actin. (**b**) Quantitative reverse transcriptase polymerase chain reaction (qPCR) results show mRNA levels of smooth muscle myosin heavy chain (SM-MHC), smooth muscle 22α, Klf4 and CaMKIIδ in the contra uninjured and injured carotid arteries 7 days after injury. (**c**) Complimentary sequences between rat CaMKIIδ 3′UTR and rat miR-30 family members. The expressions of miR-30 family members and miR-145 were compared between injured and uninjured carotid arteries and U6 expression was measured and used for normalization. Values shown are mean±S.E.M., n = 3 pairs and *p < 0.05 **p < 0.01 analyzed by paired *t-test.*

Our laboratory first reported that increased expression of CaMKIIδ_2_ (or “CaMKIIδ_C_”) protein expression in VSM promotes proliferation, migration^10^. CaMKIIδ_2_ appears to be required for vascular remodeling following injury^12^,^26^. To identify mechanisms regulating CaMKIIδ_2_ expression, we performed balloon angioplasty injury on rat carotid arteries and examined expression of CaMKIIδ_2_ protein compared to mRNA 7 days after injury (Fig. 1). Consistent with previous studies, CaMKIIδ_2_ protein expression in the left injured carotid artery was increased approximately 2-fold compared to the contralateral uninjured carotid artery (Fig. 1a), while expression of smooth muscle contractile phenotype markers calponin and smooth muscle myosin heavy chain (Myh11), were markedly decreased. mRNA expression of contractile phenotype markers, including Myh11 and transgelin (Tagln; or “sm22α”) was decreased 7 days after injury while the smooth muscle synthetic phenotype marker KLF4 mRNA was increased, indicating the phenotype switch which precedes VSM proliferation and migration leading to neointima formation^2^ (Fig. 1b). Importantly, CaMKIIδ_2_ mRNA was unchanged following vascular injury, indicating that injury-induced regulation of CaMKIIδ protein expression in VSM is post-transcriptional.

### The expression of miR-30 family members is reduced in dedifferentiated vascular smooth muscle

**Figure 2:**
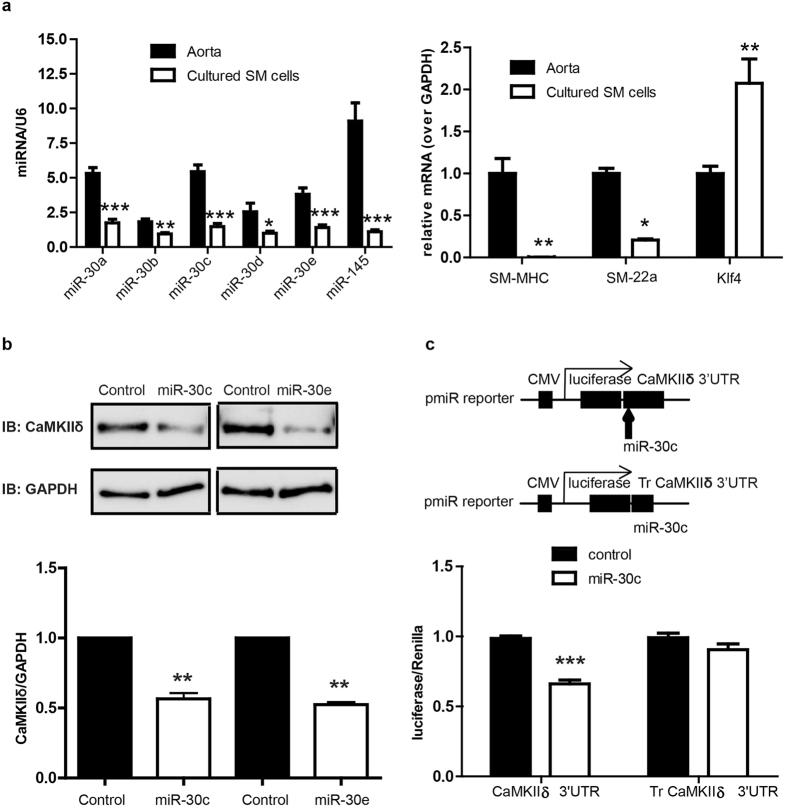
MiR-30 inhibits CaMKIIδ expression by targeting CaMKIIδ 3′UTR in primarily cultured smooth muscle cells. Rat aorta medial layers were enzymatically dispersed and primarily cultured for 3–7 passages. (**a**) The miRNA and mRNA levels of miR-30a, miR-30b, miR-30c, miR-30d, miR-30e, SM-MHC, SM-22α and Klf4 were measured by qPCR in aorta and cultured smooth muscle cells. Data were normalized over U6 (miRNA) or GAPDH (mRNA) (n = 3 aorta and 5 cultured SM cells). (**b**) miR-30c mimic or miR-30e (Invitrogen) (0.2pmol) was eletroporated into primarily cultured SM cells (1 million) and the expression of CaMKIIδ and GAPDH was analyzed by Western blot 3 days post-electroporation. (**c**) Full length of CaMKIIδ 3′UTR or truncated CaMKIIδ 3′UTR was introduced into pmiR reporter vector (Invitrogen). Full length CaMKIIδ 3′UTR reporter or truncated CaMKIIδ 3′UTR reporter as well as miR-30c and renilla were transfected into HEK293 cells and luciferase activity was measured 3 days after transfection. Values are shown as mean +/- S.E. M., n ≥ 4 and analyzed by two-way ANOVA or *t-test*. *p < 0.05 **p < 0.01 and *** *p* < 0.001.

MicroRNA is a class of small non-coding RNA which regulates the expression of target genes by either inducing mRNA decay or mediating translational repression^27^. Based on *in silico* analysis of the rat CaMKIIδ 3′UTR sequence (TARGETSCAN), a miR-30 family binding sequence is predicted (^+2036^UGUUUACA^+2043^) (Fig. 1c). This target sequence is highly conserved across species, including human (not shown). All miR-30 members were expressed in uninjured carotid medial VSM with miR-30c being the most abundant (Fig. 1d). 7 days post vascular injury, expression of all miR-30 family members was significantly reduced in medial wall VSM by 60–80%. As expected, expression of miR-145, a well-known marker and regulator of the smooth muscle contractile phenotype^23^ decreased dramatically following vascular injury and de-differentiation of VSM. Collectively, miR-30 family members are expressed at a level comparable to miR-145. Similarly, comparing miR-30 expression in differentiated VSM from intact aorta medial layers to synthetic phenotype cultured aortic medial VSM cells confirmed reduced expression of miR-30a-e in de-differentiated cells by 30–70%. (Fig. 2a). As positive controls, miR-145, SM-MHC, and SM-22α were all expressed at lower levels, and KLF4 at higher levels, in cultured VSM compared to fully differentiated aortic VSM.

### Overexpression of miR-30 reduces CaMKIIδ expression by targeting CaMKIIδ mRNA 3′UTR and inhibits VSMCs proliferation

Based on reciprocal expression dynamics between CaMKIIδ and miR-30 in VSM *in vivo* in response to vascular injury, we tested the hypothesis that CaMKIIδ is in fact an endogenous target of miR-30 and its expression is regulated by miR-30. In primarily cultured rat VSM cells, CaMKIIδ_2_ expression was inhibited approximately 50% after introduction of miR-30c or miR-30e mimics introduced by electroporation (Fig. 2b). Next, we employed a luciferase reporter assay to determine if miR-30 directly interacts with CaMKIIδ 3′UTR, and exert its inhibitory regulation. A full-length CaMKIIδ 3′UTR construct and truncated CaMKIIδ 3′UTR construct without the predicted miR-30 seed sequence (^+2036^UGUUUACA^+2043^) were cloned into a luciferase reporter vector (Fig. 2c). The reporter constructs were transiently transfected into HEK293 cells along with miR-30c mimic or a miR control. The full-length CaMKIIδ 3′UTR luciferase intensity was reduced 30% with addition of miR-30c compared to control. However, miR-30c had no effect on the truncated CaMKIIδ 3′UTR luciferase activity, suggesting miR-30c directly interacts with CaMKIIδ 3′UTR.

**Figure 3:**
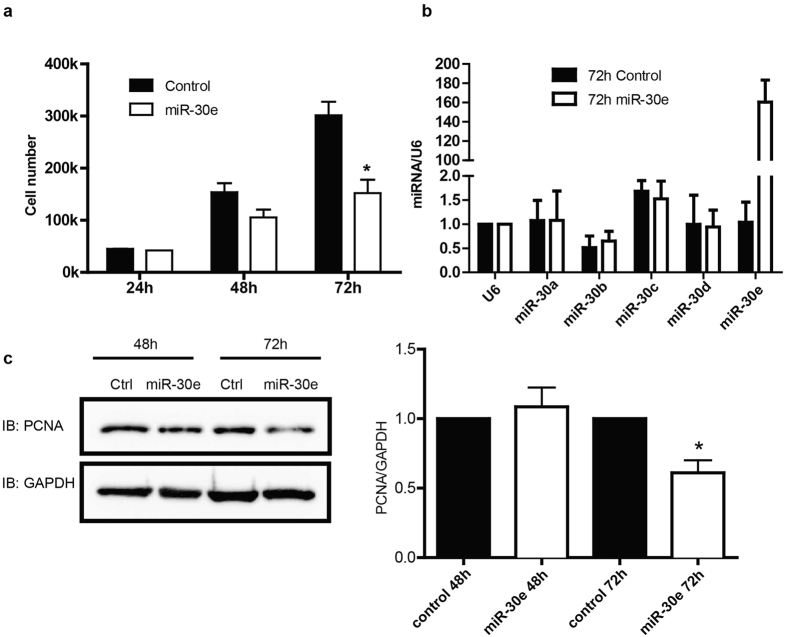
MiR-30 inhibits vascular smooth muscle cell proliferation. miR-30e mimic (0.2 pmol) was eletroporated into cultured vascular smooth muscle cells (50,000). (**a**) Cell numbers were counted at 24 h, 48 h and 72 h post eletroporation to monitor cell growth and proliferation. (**b**) The level of miR-30 family members was tested using qPCR 72 h after eletroporation. (**c**) Cell lysates were immnuoblotting for PCNA and GAPDH and quantified data were normalized over GAPDH. Values shown are mean±S.E.M., n = 3 pairs and *p < 0.05 ***p < 0.001 analyzed by two-way ANOVA.

Because CaMKIIδ is reported to promote VSM cell proliferation^10^,^12^,^26^,^28^ we tested the functional effects of miR-30 overexpression on serum-stimulated VSM cell growth. Induction of miR-30e mimic into cultured VSM significantly attenuated VSM cell growth rate after 72h by 50% compared to controls where the doubling time was 24 hr (Fig. 3a). miR-30e over-expression at 72 hr was confirmed by qPCR (Fig. 3b). miR-30e transduction had no effects on VSM apoptosis (data not shown) but significantly inhibited PCNA expression after 72 hr, a marker of active cell proliferation (Fig. 3c).

### CaMKIIδ_2_ is involved in miR-30 mediated attenuation of VSM cell proliferation

**Figure 4:**
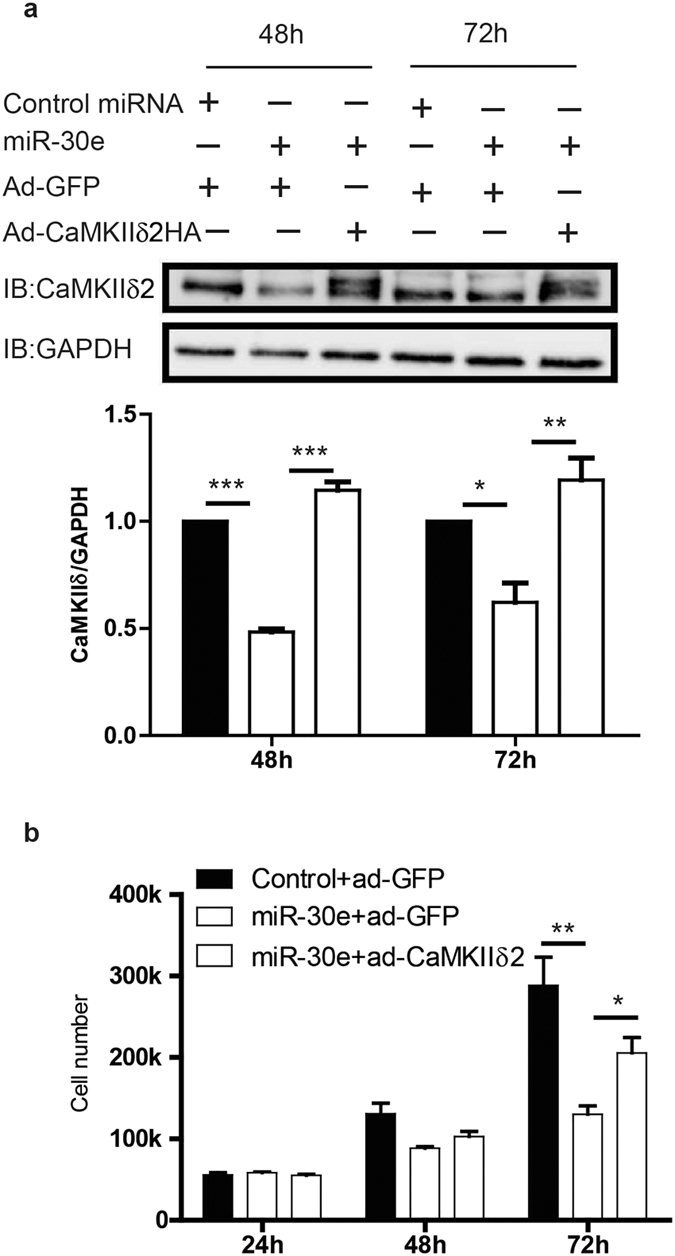
Overexpression of CaMKIIδ partially rescues VSM proliferation inhibition by miR-30e. Cultured VSM cells were eletroporated with scrambled miRNA or miR-30e mimic (0.2 pmol) and after 24 h, adeno-virus encoding CaMKIIδ2HA or GFP (2.5 MOI) was utilized to infect electroporated VSM cells for CaMKIIδ rescue. (**a**) Cell lysates were analyzed at 48 h and 72 h post eletroporation by western blot using specific antibodies for CaMKIIδ and GAPDH. Quantified data were normalized over GAPDH. (**b**) VSM Cell growth were examined by counting cell number 24 h, 48 h, and 72 h post electroporation. Values are shown as mean ± S.E.M., n = 3 and *p < 0.05, **p < 0.01 ***p < 0.001 analyzed by two-way ANOVA.

Because miRs typically have multiple mRNA targets we determined to what extent CaMKIIδ protein expression could rescue miR-30e induced inhibition of VSM proliferation. In these experiments we titrated adenoviral transduction of a wild-type CaMKIIδ_2_ construct into VSM overexpressing the miR-30e mimic such that CaMKIIδ expression after 48 and 72 hr was rescued to control levels (Fig. 4a; top panel). As shown above, miR-30e overexpression slowed VSM cell growth by 72 hr (Fig. 4b). Re-expression of CaMKIIδ_2_ to a physiological level significantly rescued VSM cell proliferation. These experiments confirm CaMKIIδ as a target of miR-30 and mediator of miR-30 induced inhibition of cell proliferation.

### Overexpression of miR-30c dramatically inhibits neointima formation

**Figure 5:**
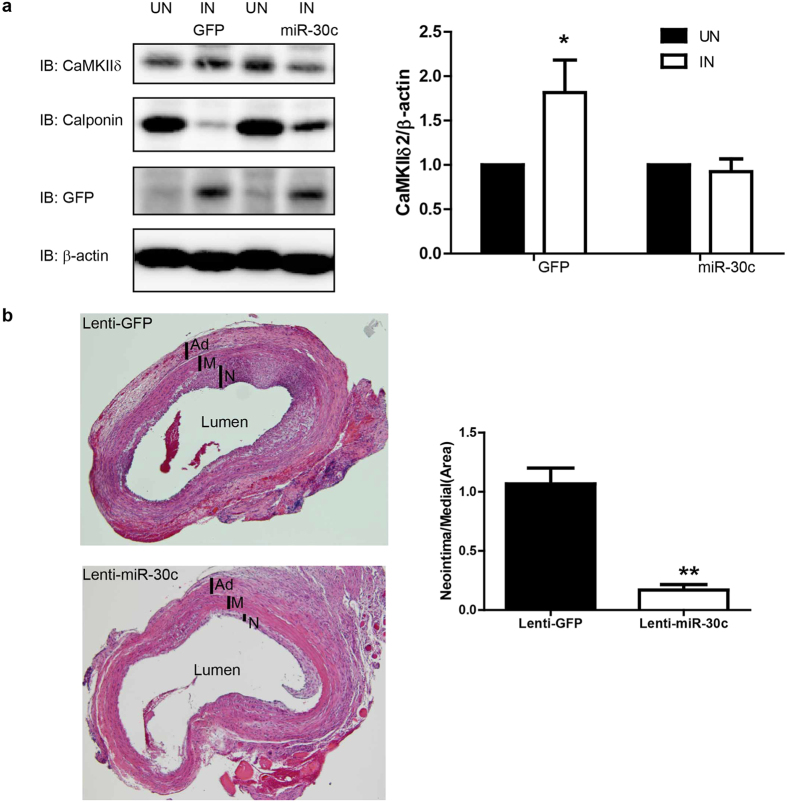
Overexpression of miR-30c prevents injury induced increase of CaMKIIδ and attenuates neointima formation. Rat carotid artery was balloon injured and locally infected with lentivirus encoding GFP and miR-30c. After 14 days, immunoblotting and histochemistry were employed for protein and morphology analysis. **(a**) The protein level of CaMKIIδ, calponin, GFP and β-actin was tested in uninjured and injured carotid arteries. Quantified data were normalized over β-actin and shown as mean ± S.E.M., n = 3. *p < 0.05 by paired *t-test* comparing CaMKIIδ expression between injured (IN) and uninjured (UN) arteries. (**b**) Injured carotid artery was fixed followed with sectioning (8μm) and staining with hematoxylin and eosin. The areas of medial layer and neointima were measured and quantified. Data shown are the ratio of neointima over medial layer, and presented as mean ± S.E.M., n = 3. ** indicates *p* < 0.01 by unpaired *t-test.*

Injury-induced neointima hyperplasia is mainly determined by VSM and/or VSM progenitor proliferation^29^,^30^,^31^. Since we demonstrated that miR-30 significantly inhibited VSM proliferation *in vitro*, in part by targeting CaMKIIδ expression, we tested effects of ectopic miR-30 expression on CaMKIIδ expression and vascular remodeling *in vivo* following rat carotid artery balloon injury. miR-30c, the most abundant miR-30 family member *in vivo*, was introduced into the medial wall by lenti-viral transduction immediately after carotid injury in order to rescue injury-induced miR-30 downregulation. GFP was also encoded by both control and miR-30c lenti-virus. GFP protein expression 2 weeks following injury was comparable between control and miR-30c transduced vessels indicating similar lenti-viral infection efficiencies (Fig. 5a). As shown previously, CaMKIIδ_2_ expression was up-regulated approximately 2-fold following vascular injury in controls. Importantly, overexpression of miR-30c significantly inhibited injury-induced CaMKIIδ_2_ up-regulation (Fig. 5a). Balloon injury followed immediately by intraluminal control lenti-virus resulted in growth of an asymmetric neointimal layer, similar to previously reported results^11^,^12^,^32^ (Fig. 5b, top panel). Administration of a lentivirus encoding a pre-miR-30c construct dramatically inhibited neointima formation 14 days after injury comparing to control (Fig. 5b, lower panel). Quantifying neoinitmal areas as a ratio to medial wall area indicated a greater than 80% decrease in neointima formation following miR-30c overexpression.

## Discussion

Accumulating evidence demonstrates the importance of signaling by the multifunctional protein kinase CaMKIIδ-isoform in regulating VSM cell proliferation and migration, contributing to vascular remodeling during restenosis and hypertension^5^,^33^. We have demonstrated changes in the relative content of co-expressed CaMKIIδ and –γ isoforms in VSM associated with phenotype switching following vascular injury or cell culture; changes that have important functional consequences. In particular, increased relative expression of CaMKIIδ is critical for promoting VSM proliferation, migration and neointimal remodeling in response to injury^5^,^34^,^35^ and decreased expression of CaMKIIγ is permissive for this process^11^. Our results indicating increased CaMKIIδ protein expression without increased mRNA expression in ligated mouse carotid artery^11^ and balloon-injured rat carotid arteries (Fig. 1a,b), indicate that post-transcriptional regulation of CaMKIIδ expression could be a critical factor in promoting injury-induced vascular remodeling.

Previous *ex vivo* studies using portal veins from Dicer knockout mice^22^ and *in vitro* studies using A10 cells, a transformed rat aortic smooth muscle cell line, indicated the potential for miR-145-dependent regulation of CaMKIIδ protein expression^23^. In the present studies we focused on CaMKIIδ regulation by miR-30 family members which are predicted to bind to a species-conserved sequence in the CaMKIIδ 3′-UTR. Using well-established aortic VSM primary cell culture and vascular injury models, we demonstrated; 1) an inverse correlation between CaMKIIδ protein and miR-30 family expression in VSM cells upon phenotype switching *in vitro* and in response to vascular injury *in vivo*; 2) regulation of CaMKIIδ expression and cell proliferation by miR-30 in cultured VSM with magnitude changes comparable to those observed *in vivo* following vascular injury; and 3) prevention of CaMKIIδ upregulation with inhibition VSM cell proliferation and neointima formation *in vivo* following ectopic miR-30 expression. The significance of the latter experiment is two-fold; confirming miR-30 dependent regulation CaMKIIδ *in vivo* and suggesting the therapeutic potential for miR-30, at least in the context of restenosis.

Because the synthetic phenotype cultured VSM cells expressed low levels of miR-30 family members, and effective miR-30 silencing would require siRNAs targeting all of the redundant family members, we relied on gain-of-function approaches to test if CaMKIIδ was, in fact, a direct target for miR-30. miR-30c and miR-30e mimics significantly reduced CaMKIIδ protein expression by about 50% and inhibited activity of a luciferase-CaMKIIδ 3′-UTR construct by 30%. Deletion of the putative miR-30 binding sequence in the CaMKIIδ 3′-UTR construct abrogated the effects of miR-30, supporting the hypothesis that CaMKIIδ is a direct target for miR-30 in VSM. These magnitude changes are comparable to those previously observed in A10 cells using miR-145^23^ and the partial effects in both studies could be due redundant effects of the miR-30 and miR-145 species. Alternatively, the strong CMV promoter engineered into the reporter plasmid may simply overwhelm modulatory effects of the miRs on construct stability.

Our previous studies using alternative approaches to silence CaMKIIδ expression with siRNA, or inhibit kinase activity with a virally transduced kinase-negative CaMKIIδ mutant, resulted in repressed synthetic phenotype VSM functions, including proliferation and migration^10^,^12^. In the present studies, miR-30 overexpression decreased CaMKIIδ expression and inhibited VSM proliferation *in vitro*, an effect that could be partially rescued by re-expression of CaMKIIδ to physiological levels. Partial rescue suggests there are other targets of miR-30 in addition to CaMKIIδ that could be involved in miR-30 induced inhibition of VSM cell proliferation. CaMKIIδ has been reported to regulate VSM proliferation through signaling pathways that result in suppression of p53 and p53-targets including CDKN1 (p21), a cell cycle inhibitor^26^. miR-30 family members have been reported to function as tumor suppressors by inhibiting cell proliferation, invasion and inducing apoptosis through different mechanisms, such as targeting BCL9 and repressing Wnt signaling^36^. It is a general feature of microRNA-dependent regulation that multiple transcripts are targeted^18^,^19^. Given the diversity of pathways impinging on cell cycle control, multiple signaling pathways may also be regulated in VSM by miR-30 and contribute to its net inhibitory effects on VSM proliferation. This could include other components of Ca^2+^-dependent signaling pathways including TRPC6, calcineurin, and NFATC3^37^, all of which have been implicated in control of VSM synthetic phenotype functions^38^.

MiR-30 family member expression is significantly decreased following balloon angioplasty injury of rat carotid arteries coincident with CaMKIIδ upregulation and neointimal vascular remodeling. Importantly, lentiviral transduction of miR-30 *in vivo*, immediately following injury, largely prevented subsequent CaMKIIδ upregulation and strongly inhibited VSM cell proliferation and neointimal remodeling. Neointima formation is a balance between VSMC proliferation and apotosis, and our data does not exclude the possibility that miR-30 regulates VSMC apoptosis *in vivo*, thus contributing to its inhibitory effect on the neointima formation. Recent studies have also pointed to the potential contribution of VSM progenitor cells in vascular remodeling and disease^31^. Moreover, it has been reported that miR-30 regulates epithelial to mesenchymal transition (EMT) and mesenchymal to epithelial transition (MET) in various tumor cells^39^,^40^. Thus, it needs to be studied if miR-30 interferes with the activation or promotes differentiation of vascular progenitor cells, resulting in the inhibition of neointima hyperplasia.

Collectively, in differentiated carotid arteries (Fig. 1) and aorta (Fig. 2) miR-30 family members are expressed at levels comparable to miR-145 which is considered a high abundance microRNA associated with and regulating the differentiated VSM phenotype^23^. Upon vascular injury or culture of the VSM cells, total miR-30 levels decrease by approximately 75%, similar in magnitude to the decrease in miR-145 and coincident with the switch to a VSM cell synthetic phenotype. miR-145 over-expression has also been shown to have a strong effect on VSM phenotype and neointima formation^23^. We expect that miR-30, miR-145 and other miRs act collectively to regulate the VSM phenotype switch which involves changes in expression of hundreds of proteins.

Previous studies have suggested coordinate down-regulation of miR-30 family members in skeletal muscle as a function of de-differentiation or dystrophic disease^41^, in cardiac muscle as a function of hypertrophy heart failure^42^ or atrial fibrillation^43^, in human thoracic aortic dissection^44^, and in cardiac and VSM cells following ER stress^45^,^46^. Mechanisms underlying coordinate regulation of miR-30 family members are not known. Interestingly, miR-30 family members are encoded on 3 different chromosomes in clustered pairs: miR30a and –c2 on human chromosome 6, miR-30b and-d on chromosome 8, and mir30e and –c1 on chromosome 1. A recent report indicates human coordinate regulation of several miR-30 family members by Mef2 in skeletal muscle^47^. Additionally, when Shi *et al*. induced apoptosis using TGF-β in podocytes, miR-30 expression was observed to be down-regulated in a Smad2-dependent manner^48^.

In summary, we have shown that elevated CaMKIIδ protein and attenuated miR-30 expression in dedifferentiated/synthetic vascular smooth muscle cells *in vitro* and *in vivo*. Overexpression of miR-30 significantly inhibits CaMKIIδ protein expression in cultured VSMCs. Furthermore, overexpression of miR-30 impairs cultured VSM cell proliferation, and CaMKIIδ rescue in the presence of miR-30 partially, but significantly recovers VSM cell growth. Lenti-viral delivery of miR-30 to the injured carotid artery walls dramatically inhibits cell proliferation in the medial walls and neointima hyperplasia. CaMKIIδ, the predominant isoform in cardiac muscle has also been studied extensively in the context of cardiac physiology and pathophysiology and is a critical factor in development of heart failure^49^,^50^,^51^. Based on these reports, the current studies, and one report that identified miR-30b as a regulator of CaMKIIδ expression in cardiomyocytes, it is reasonable to propose that dysregulation of a miR-30/CaMKIIδ axis in both VSM and heart might contribute to diverse cardiovascular diseases.

## Methods

### Materials

Polyclonal antibody against CaMKIIδ was raised in rabbit as described previously^52^,^53^. The antibodies for β-actin, GAPDH, and Calponin were purchased from Sigma. MiRNA mimics were purchased from Invitrogen and lenti-viral plasmid expressing miR-30c was purchased from System Biosciences.

### VSM cell dispersion and culture

Primarily cultured VSM cells were obtained from rat aorta as described previously^34^. All animal usage for obtaining primary rat VSM cells was reviewed and approved by IACUC (14-01006) and the Albany Medical Center Institutional Animal Care and Use Committee. All animal experiments and procedures were carried out in accordance with the approved guidelines. Briefly, Sprague-Dawley rats (150–200g) (Taconic) were sacrificed with CO_2_ and medial layers of aorta containing VSM cells were mechanically isolated from adventitial layer and endothelial layer. Medial VSM layers were enzymatically digested with collagenase and elastase and dispersed VSM cells were cultured in DMEM/F12 (Gibco) supplemented with 10% fetal bovine serum (GE healthcare) and 1% penicillin/streptomycin (GE healthcare). All experiments were carried out with VSM cells from passage 3 to 6.

### Lenti-virus production

To transduce miR-30 into the common carotid artery wells *in vivo*, we produced lenti-virus encoding miR-30c using HIV-based pPACKH1 packaging system (System Biosciences, CA, USA). In brief, HEK293 FT cells (Invitrogen) under passage 15 were grown in DMEM (Gibco) supplemented with 10% fetal bovine serum (GE healthcare) and 1% penicillin/streptomycin (GE healthcare) on 100 mm petri dish. When the confluency reached 80–90%, the culture media were replaced with DMEM supplemented with 10% serum and cells were transfected with pPACKH1-GAG (3.3 μg), pPACKH1-REV (3.3 μg), pVSV-G (3.3 μg) and lenti-viral plasmid encoding miR-30c or empty lenti-viral vector (2 μg) using *Trans*IT^®^-2020 (Mirus Bio, WI, USA) (36 μl). After 16 h incubation, the culture media were changed to DMEM with 0.01% fetal bovine serum and 1% penicillin/streptomycin, followed by 24 h incubation for virus production. The media containing mature lenti-virus were harvested and concentrated.

### Luciferase reporter assay

To study if miR-30 binds to CaMKIIδ 3′UTR, full-length of CaMKIIδ 3′UTR (Accession: NM_012519.2, +1825 to +2300) and truncated CaMKIIδ 3′UTR (+1825 to +2035) without miR-30 binding site (^+2036^UGUUUACA^+2043^) were cloned into pMIR-REPORT™ miRNA Expression Reporter Vector (Invitrogen). HEK293 cells were grown on 35mm petri dish and transiently transfected with combinations of CaMKIIδ 3′UTR luciferase reporter (2 μg), TK-*Renilla* (0.1 μg) and miRNA mimic control (0.4 nmole); CaMKIIδ 3′UTR luciferase reporter (2 μg), TK-*Renilla* (0.1 μg) and miR-30c mimics (0.4 nmole) or trucanted CaMKIIδ 3′UTR luciferase reporter (2 μg), TK-*Renilla* (0.1 μg) and miR-30c mimics (0.4 nmole) by using lipofectamine2000 (Invitrogen) and incubated for total 72 h prior to cell lysis. Luciferase reporter activity was determined by measuring firefly luciferase signal intensity normalized over the TK-*Renilla* intensity using the Dual Luciferase Assay System (Promega).

### Rat carotid artery balloon injury

Rat carotid artery balloon injury has been described previously^54^. Male Sprague-Dawley rats (400–430 g) (Taconic) were anaesthetized with the mixture of ketamine (70 mg/kg) and xylazine (4.6 mg/kg) via intraperitoneal injection. After a midline cervical incision and blunt muscular tissues separation, left common carotid artery was dissected until the exposure of the bifurcation of the internal and external carotid arteries. The external carotid artery was completely ligated and blood flow was ceased by two vessel clips on distal internal carotid artery and proximal common carotid artery. A small arteriotomy was made between the bifurcation and ligation suture on the external carotid artery, followed by inserting a 2F Fogarty embolectomy catheter into the common carotid artery through the small cut. The catheter was then inflated under the pressure of 1.5–1.8 ATM, followed by pulling the inflated catheter through the common carotid artery for 3 times. After injury, a complete ligation was performed between the bifurcation and the small cut and then the blood flow was rebuilt up by releasing two vessel clips. For experiments delivering lenti-virus encoding miR-30c and control lenti-virus to the injured segment of the common carotid artery, prior to the second complete ligation, concentrated lenti-virus media were injected into and incubated in the injured common carotid artery lumen for 30min, followed with removal of virus media from the artery lumen. All animal usage for rat carotid artery angioplasty injury was reviewed and approved by IACUC (12-07004) and the Albany Medical Center Institutional Animal Care and Use Committee. All animal experiments were carried out in accordance with the approved guidelines.

### MiRNA mimics overexpression

VSM cells (1 × 10^6^) were harvested and electroporated with either miR-30c/e mimics (0.1 nmol) or miRNA mimic control (0.1 nmole) (Invitrogen) using the Amaxa Nucleofector system (Lonza) and the VSM cell-specific D33 program (Lonza Amaxa). After electroporation, cells were onto 4 × 60mm petri dishes and total RNA and protein were extracted 48 h and 72 h post-electroporation.

### Western blot and Quantitative PCR

VSM cells were washed with DPBS twice and lysed with either 3× Laemmli sample buffer for Western blot or Trizol (1 ml) (Invitrogen) for RNA extraction. Uninjured or injured common carotid arteries were harvested and homogenized either in RIPA buffer for Western blot and Trizol (1 ml) for total RNA extraction. Cell lysates or tissue lysates were heated at 95 ^o^C for 5 min and resolved using 10% SDS-PAGE, followed by transferred onto nitrocellulose membranes (GE healthcare). The membranes were blocked with 5% non-fat milk or BSA in Tris-buffered saline with 1% Tween 20 for 30 min and incubated with primary antibody for 1 h at room temperature and 4 ^o^C overnight, followed by secondary antibody incubation for 1 h. After extensive washing with TBST, the membranes were incubated with the SuperSignal chemiluminescent substrate for 5 min and gel images were digitalized by Fuji LAS4000.

### H&E staining

Balloon injured common carotid arteries were harvested from euthanized animals and fixed overnight in 4% buffered paraformaldehyde (Fisher Scientific). Fixed vessels were then dehydrated and embedded in paraffin blocks. Blocks were cut into 5 μm sections and recovered on Superfrost Plus slides (Fisher Scientific). Sections were cleared with Xylene (Cardinal Health) and rehydrated in sequential rinses of 100%, 80% and 70% ethanol. Sections were then stained with hematoxylin and eosin (H&E) (Thermo Scientific). Sections were examined at 10x magnification on an Olympus BX51 microscope.

### Statistical analysis

Quantitative data were presented as mean ± S.E.M. Student’s unpaired or paired t test was performed in the comparison between two groups. Comparisons among multiple groups were carried out using two-way ANOVA with Bonferroni post hoc test. All statistical analysis was performed using GraphPad Prism5 program. “n” indicates the number of independent experiments and significance is presented as, *,** and *** indicate p < 0.5, p < 0.01 and p < 0.001 respectively.

## Additional Information

**How to cite this article**: Liu, Y. F. *et al*. MicroRNA-30 inhibits neointimal hyperplasia by targeting Ca^2+^/calmodulin-dependent protein kinase IIδ (CaMKIIδ). *Sci. Rep.* **6**, 26166; doi: 10.1038/srep26166 (2016).
